# Predicting and verifying outcome of *Tripterygium wilfordii Hook F*. based therapy in rheumatoid arthritis: from open to double-blinded randomized trial

**DOI:** 10.1038/srep09700

**Published:** 2015-04-15

**Authors:** Miao Jiang, Qinglin Zha, Chi Zhang, Cheng Lu, Xiaoping Yan, Wanhua Zhu, Wei Liu, Shenghao Tu, Liping Hou, Chengwu Wang, Wandong Zhang, Qinghua Liang, Bing Fan, Jiangping Yu, Weidong Zhang, Xinru Liu, Jing Yang, Xiaojuan He, Li Li, Xuyan Niu, Yan Liu, Hongtao Guo, Bing He, Ge Zhang, Zhaoxiang Bian, Aiping Lu

**Affiliations:** 1 Institute of Basic Research in Clinical Medicine, China Academy of Chinese Medical Sciences, Beijing 100700, P.R. China; 2 School of Chinese Medicine, Hong Kong Baptist University, Kowloon Tong, Kowloon 999077, Hong Kong; 3 School of Computer, Jiangxi University of Traditional Chinese Medicine, Nanchang 330004, P.R. China; 4 Department of Rheumatism, Beijing China-Japan Hospital, Beijing 100621, P.R. China; 5 Nantong Liangchun Rheumatology Hospital, Nantong, Jiangsu 226009, P.R. China; 6 Department of Rheumatism, The first affiliated hospital of Tianjin University of TCM, Tianjin 300193, P.R. China; 7 Department of Rheumatism, Hubei Tongji Hospital, Wuhan, Hubei 430030, P.R. China; 8 Shanxi Taiyuan Rheumatology Hospital, Taiyuan 030006, Shanxi, P.R. China; 9 Department of Rheumatism, The first hospital affiliated to Jilin Changchun University of TCM, Changchun, Jilin 130021, P.R. China; 10 Department of Rheumatism, The first hospital affiliated to Anhui University of TCM, Hefei, Anhui 230031, P.R. China; 11 Department of Rheumatism, Hunan Xiangya Hopital, Changsha, Hunan 410008, P.R. China; 12 Department of Rheumatism, Affiliated Hospital to Shandong University of TCM, Jinan, Shandong 250011, P.R. China; 13 Department of Rheumatism, The first hospital affiliated to Jiangxi University of TCM, Nanchang 330020, Jiangxi, P.R. China; 14 College of Pharmacy, The Second Military Medical University, Shanghai 200433, P.R. China; 15 Department of Rheumatism, The first hospital affiliated to Henan College of Chinese medicine, Zhengzhou, Henan, 450000, P.R. China

## Abstract

*Tripterygium wilfordii Hook F.* (TwHF) based therapy has been proved as effective in treating rheumatoid arthritis (RA), yet the predictors to its response remains unclear. A two-stage trial was designed to identify and verify the baseline symptomatic predictors of this therapy. 167 patients with active RA were enrolled with a 24-week TwHF based therapy treatment and the symptomatic predictors were identified in an open trial; then in a randomized clinical trial (RCT) for verification, 218 RA patients were enrolled and classified into predictor positive (P+) and predictor negative (P−) group, and were randomly assigned to accept the TwHF based therapy and Methotrexate and Sulfasalazine combination therapy (M&S) for 24 weeks, respectively. Five predictors were identified (diuresis, excessive sweating, night sweats for positive; and yellow tongue-coating, thermalgia in the joints for negative). In the RCT, The ACR 20 responses were 82.61% in TwHF/P+ group, significantly higher than that in TwHF/P− group (*P* = 0.0001) and in M&S/P+ group (*P* < 0.05), but not higher than in M&S/P− group. Similar results were yielded in ACR 50 yet not in ACR 70 response. No significant differences were detected in safety profiles among groups. The identified predictors enable the TwHF based therapy more efficiently in treating RA subpopulations.

Rheumatoid arthritis (RA) is a chronic, systemic autoimmune inflammatory disorder of unknown aetiology that occurs in about 1% of the adult population, which leads to disability and premature death^1^. The management of RA includes pharmacological, non-pharmacological, invasive and surgical interventions that should be tailored to each patient's disease manifestations, such as disease activity, current symptoms, laboratory findings, and prognostic indicators. Disease-modifying antirheumatic drugs (DMARDs) are recommended as the first-line treatment for RA^2^,^3^. Immunoselective biologic agents, such as TNF inhibitors, have been introduced for RA treatment as second-line drugs^4^. However, no treatment cures RA^2^. Due to the insufficient response, adverse events, and high expense of the current pharmacological therapies, new source of drugs in RA treatment is still warranted^2^,^3^.

Tripterygium wilfordii Hook F (TwHF) is regarded as a potential source of new drugs in RA treatment^5^,^6^,^7^,^8^,^9^ for the TwHF based therapy has been proved to be able to achieve better effectiveness than DMARDS monotherapy in controlling disease activity in patients with active RA^10^,^11^. However, the lack of consensus on the effectiveness and safety evaluation of TwHF products in treating RA has limited the new drug development based on TwHF: systematic reviews presented an inconsistent or ambiguous evaluation on the efficacy of TwHF products^6^,^7^,^12^, some indicated TwHF extracts could be as effective as synthetic DMARDs^12^,^13^; others concluded these products were not recommended for RA^14^.

One major cause for this inconsistency lies in that the optimal effect of TwHF based therapy could be obtained if only the TwHF products being used to treat the right subgroup of patient defined by traditional Chinese medicine (TCM) pattern (syndrome or Zheng) classification, as claimed by TCM clinicians^15^,^16^. TCM identifies and treats the patients with corresponding TCM patterns^17^, and baseline symptoms, which are the basis of pattern differentiation, are regarded as important predictors of response.

Researchers are trying to establish various predictors of response to therapy in rheumatic diseases, which may facilitate to improved patient selection and therapeutic outcomes^18^,^19^,^20^,^21^, yet these predictors, including baseline characteristics, gene polymorphism, proteins, microRNAs, or mixed model, etc. are seldom verified in a randomized, controlled trial (RCT), although that is a must precedence to the clinical usage of these findings. Before the solid biomarkers can be conveniently and affordably used in daily clinical practice, baseline symptoms should be the most ideal predictors of response for its convenience and facility of acquire and identification.

Actually, some baseline clinical factors have been detected to be able to predict better outcome in treating rheumatic diseases with various drugs^19^,^20^,^22^,^23^. Furthermore, in our previous multicenter RCT, some symptoms, including those inquired based on TCM pattern classification theory were indicated to have potential correlation with the effect and safety of TwHF based therapy in RA patients^24^,^25^,^26^,^27^. These findings merit further study to ascertain the accurate baseline symptomatic predictors of response to the TwHF based therapy in light of TCM experiences thus to help clinicians to make evidence-based decisions which might maximize the benefits from treatment by targeting subgroups of patients most likely to respond.

Given these concerns, in this study we developed a rigorously designed two-stage clinical trial aiming at proving the impact of the baseline symptomatic predictors of response to TwHF based therapy in treating RA. The predictors was identified in the first stage open-labeled trial and then verified in the second stage double-blind and double-dummy RCT. The effectiveness and safety of this therapy in the subgroup of RA patients defined with this predictors were also assessed compared to the control therapy, combinative methotrexate (MTX) with sulfasalazine (SSZ) therapy.

## Methods

### Design Overview

The design of this two-stage trial in the management of RA was previously described^28^. The first stage trial was a 24-week open-labeled, multicenter trial and aimed at identifying the baseline symptomatic predictors of the TwHF based intervention. The second stage trail was designed as a randomized, double-blind, stratified, double-dummy, positive controlled, multicenter study and aimed at verifying the prediction of the identified symptomatic predictors as well as assessing the effectiveness and safety of the TwHF based therapy in the subgroups of RA patients defined by the predictors.

The whole study was conducted according to the Declaration of Helsinki and the International Conference on Harmonization Tripartite Guideline on Good Clinical Practice^29^. Approvals from the appropriate research ethics committees were obtained before the trials began: Ethics Committee of Institute of Basic Research in Clinical Medicine, China Academy of Chinese Medical Sciences (No. 2008NO3 for the first-stage open trial; No.2010NO6 for the second-stage RCT). All patients were asked to provide written, informed consent before participating. Trial registration no. was ChiCTR-TRC-10000989.

### Setting and Participants

In the open clinic trial, 167 patients were enrolled from March, 2008 to January, 2010. In the RCT, 218 patients were enrolled from August, 2010 to June, 2012, from eight authorized rheumatology departments in general hospitals in China (Beijing China-Japan Hospital, The first affiliated hospital of Tianjin University of TCM, Hunan Xiangya Hopital, Hubei Tongji Hospital, The first hospital affiliated to Jilin Changchun University of TCM, The first hospital affiliated to Anhui University of TCM, The first hospital affiliated to Jiangxi University of TCM, Affiliated Hospital to Shandong University of TCM) and 2 rheumatology hospitals in China (Nantong Liangchun Rheumatology Hospital, Shanxi Taiyuan Rheumatology Hospital).

The criteria for entry into the study were an age of 18 to 70 years; class I, II, or III stage RA fulfilling the criteria of the American Rheumatism Association^30^; and active disease (active RA was defined by the presence of three or more swollen joints, six or more tender joints, morning stiffness that lasted at least 30 minutes, and at least one of the following: an erythrocytese dimentation rate of at least 28 mm per hour, or an elevated serum C-reactive protein concentration of at least 20% higher than normal value). Patients were not eligible for the study if they had other important concurrent illnesses; if they were allergic to any of the study drugs; if they received other commercial or experimental biological therapies for RA, if they were women of childbearing age who were not using contraception. DMARDs other than MTX and SSZ, including hydroxychloroquine, cyclophosphamide, oral corticosteroids (≤10 mg prednisone or equivalent), were discontinued at least four weeks before the study began. Stable doses of nonsteroidal anti-inflammatory drugs were allowed.

### Randomization and Interventions

An independent clinical research coordinator (CRC) performed the randomization with a central randomization system designed by the Hospital Affiliated to Nanjing University of Chinese Medicine (Version No. 115ZD_BJ_RA_LFG001). 

The randomization number for each subject was produced by the randomization system according to the patients' disease courses. Patients were stratified based on the disease courses: within 1 years; from 1 to 3 years; longer than 3 years. Block randomization was applied in each center (the block number was 144 and the block length was 6). First, the randomization form with the basic information of the participant who passed the screening phase was transmitted by the internet randomization system/telephone, then the system would allocate the randomization number (specific ID number) based on the study design. The completion of this procedure was guaranteed by the research organization. The CRC was separated from all researchers, and the researchers didn't have any influence on enrollment or randomization. Any contact between the statistician and clinical researchers would not occur. The CRC also acted as the independent data and safety monitoring board.

**Figure 1:**
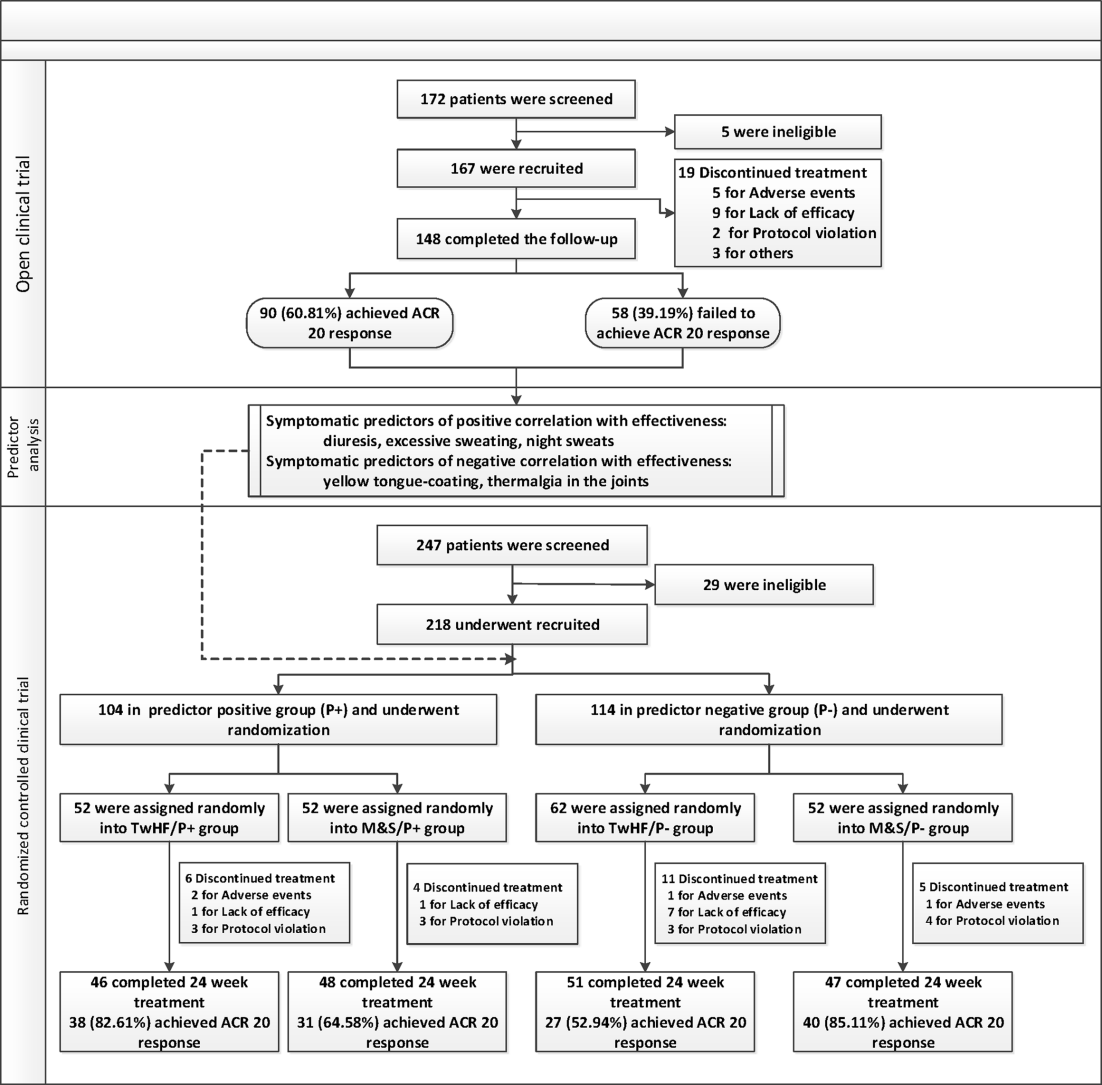
Study flow diagram.

In the open trial, all the recruited patients received the TwHF based intervention: the combination of Glucosidorum Tripterygll Totorum (GTT, Leigongteng Duogan tablets) and a Chinese herbal product Yi Shen Juan Bi pill (YSJB). Both medicines are marketed as herbal medications for RA patients, the combinative therapy is recommended by the Chinese Association of Integrative Medicine^31^. GTT tablets (provided by Jiangsu Meitong Co., Ltd. Z43020138) were prescribed at 10 mg 3 times a day after meals. YSJB (patent number: ZL200510040550; prepared by Qingjiang Co., Ltd. Z10890004; the fingerprints of YSJB is shown as Supplemetary Figure 1 to make sure the consistency of the quality of the product) was prescribed at 8 g/each time and 3 times a day after meals. Oral Votalin (Diclofenac) sustained release tablet (provided by Beijing Novartis Pharma. Ltd. Drug registration Number: X0271) was permitted for the patients with severe joint pain based on the doctor's judgment, and the dosage was 75 mg once a day. No other drugs for treating RA were permitted.

In the RCT, 218 RA patients (recruited according to the inclusion criteria set for the open trial) were enrolled. According to the symptomatic predictors identified in the open trial (referring to results part), patients were classified into predictor positive group (P+) and predictor negative group (P−). Then the 2 groups of patients went on the randomization allocation, respectively.

In both P+ and P− group, patients were randomly assigned to two subgroups, one received the TwHF based therapy as in the open trial and placebos of MTX plus SSZ therapy; the other received MTX plus SSZ therapy and placebos of TwHF based therapy. The ratio of randomization allocation to the sites was 1:1. MTX plus SSZ therapy included Methotrexate (MTX, provided by Shanghai Xinyi Pharmaceutical Co., Ltd. Drug registration Number: Z070404), 10 mg/week and Sulfasalazine (SSZ, provided by Xi'an Kangbaier Pharmaceutical Co., Ltd. Drug registration Number: Z070404), at an initial dose of 0.5 g 3 times a day in the first week, from the second week the dose was 1.0 g twice a day. All placebos were provided by the pharmaceutical factory which provided the associated drugs for ensuring the identical appearance, taste and package.

The treatment course in both stages was 24 weeks. Study visits occurred at screening, at baseline (day 1), at week 2, 4, and every 4 weeks through week 24 or withdrawal from the study.

Use of oral Votalin and other drug, the treatment course and study visits were the same as design in the open trial.

### Outcomes and follow-up

In both stages of the trial, the primary effectiveness endpoint is the ACR 20 responsive rate^32^ at week 24, which was defined according to the ACR definition of a 20% improvement. Secondary effectiveness endpoints include the ACR50 and ACR70 responses and the individual components of the ACR. Symptoms, joint function, physical exam, laboratory tests, and patient-reported outcomes, including the scores of the Health Assessment Questionnaire (HAQ) were also noted^33^. All of these endpoints over time in the study were analyzed as exploratory endpoints. Safety endpoints include adverse events (AEs), serious AEs (SAEs), and laboratory abnormalities.

The symptoms and signs were evaluated by two independent assessors who had no knowledge of the patient's treatment assignment. Given that in TCM practice, clinicians pay more attention to symptoms and signs which are described in detail, the TCM symptoms are included in this study, yet the ones easy to be confused are omitted, such as pulse manifestation.

In TCM, the defining symptoms and signs of a patient are all those deviations in all bodily functions and appearances from the norms of Chinese medicine as gathered by the four examinations. The four examinations are looking, listening-smelling, palpating, and questioning. Thus those symptoms and signs were obtained both from patient-reported outcomes and physicians. They are terminologically standardized by China TCM academics who published *Traditional Chinese Medicine Dictionary (In Chinese, People's Health Press, 2005)*, and also in *the English-Chinese Chinese-English Dictionary of Chinese Medicine (Hunan Science & Technology Press, 1995)* and *WHO international standard terminologies on traditional medicine in the western pacific region (World Health Organization, 2007)*. By referring to those term standards, we defined all the symptoms and signs investigated in this study, which were shown in Supplementary Table 2.

To make sure to get objective observation on those symptoms and signs, they were firstly explained to patient by investigators to guarantee the patient can fully understand each item, and then were recorded by clinicians who performed the inquiry and explanation using a specific designed symptom questionnaire.

### Statistical Analysis

Sample size was calculated using PASS (version 11.0, NCSS, LLC, Kaysville, Utah, USA) software. For open clinical trial: concerning the aim of the open trial, a logistic regression of a binary response variable (Y) on a binary independent variable (X) was adopted^34^. The sample size was of 149 observations (of which 50% are in the group X = 0 and 50% are in the group X = 1) achieves 80% power at a 0.050 significance level to detect a change in Prob (Y = 1) from the baseline value of 0.532 to 0.750.

Sample size calculation for RCT: the RCT was designed to detect differences of TwHF based therapy in treating patients in the predictor positive group (P+) versus in predictor negative group (P−). Sample size was calculated to detect differences in the primary end point with greater than 80% power at a 2-side level of significance of 0.05 between the two groups. Given the two groups of patients are belonging to two separate subgroups, the term for such a comparison is a test of interaction. Therefore, we designed a control group using a standard combination therapy (MTX plus SSZ, M&S). According to previous studies^35^ and the first stage trial, the ACR 20 responsive rate is supposed to be in this order: TwHF/P+ group > M&S > TwHF/P− group assuming the effective rates of MTX plus SSZ are insusceptible with the indicators (similar effective rate in P+ and P− group). Thus the sample size calculation for RCT was calculated to detect differences of TwHF based therapy versus M&S in RA patients. The ACR20 responsive rate was set to be 58% of M&S^36^, and 87.7% of TwHF/P+ group (based on the predictive results from the open trial data): P1 = 0.877 (TwHF based therapy group proportion | H1), P2 = 0.58 (control group proportion). Thus it was estimated that group sample sizes was 33 in each group. In the open trial, there were 57 out of 147 patients identified as in I+ group (38.5%), thus it was estimated in the RCT, at least 33/38.5% = 86 patients would need to be enrolled to assure 33 patients assigned in P+ group under each intervention. Given a dropout rate for follow-up as 20%, the sample size should be 214 cases in total.

For predictor determination analysis, the correlation between ACR 20 response at 24 week and baseline symptoms was analyzed by univariate analysis. The 30 TCM symptoms (including tongue appearances) at baseline were screened using chi square test, the symptoms significantly correlated with ACR20 response (*P* < 0.2) were selected and entered in a multivariate model. In the multivariate analysis, partial lease square (PLS) method was employed to establish the predictive model for ACR 20 response by baseline symptoms, ACR20 response was adopted as dependent variable, and TCM symptom was independent variable, factor number was 2 in the model. The results were expressed as weight value, the symptoms with highest weight absolute values were identified as the predictors.

For assessment, in the open clinical trial, patients were analyzed according to the open-label study^37^,^38^; in the RCT, all statistical tests were based on a 2-sided 5% level of significance using SAS software (version 9.3, SAS Institute, Cary, North Carolina, USA).

In the baseline information analysis, for dichotomous variables, chi square test was used; for ordered variables, Wilcoxon Rank test was used; for continuous variables, analysis of variance and Wilcoxon Rank test were used.

Effectiveness was assessed in the patients with 100% drug compliance [Drug compliance is defined as: (Distributive dosage of study drug – retrieved dosage of study drug)/medical ordered dosage of drug ×100%]. For the primary effectiveness endpoint analysis, firstly the ACR 20 responsive rate at week 24 was compared between TwHF based therapy and M&S in P+ and P− group, respectively. For second effectiveness endpoint and safety analysis, the comparisons of proportions (for dichotomous variables) between TwHF based therapy and M&S in the same subgroups were performed using chi square test. In respect to the comparison of TwHF based therapy effectiveness between P+ and P− groups, the method introduced for comparing two estimates of the same quantity derived from separate analysis^39^ were adopted.

Safety was assessed in patients who received one or more drug doses and had one or more post baseline safety assessments. The frequencies of AEs were calculated, and the incidences were compared by chi square test and exact probability methods.

## Results

### Study Process

**Figure 2:**
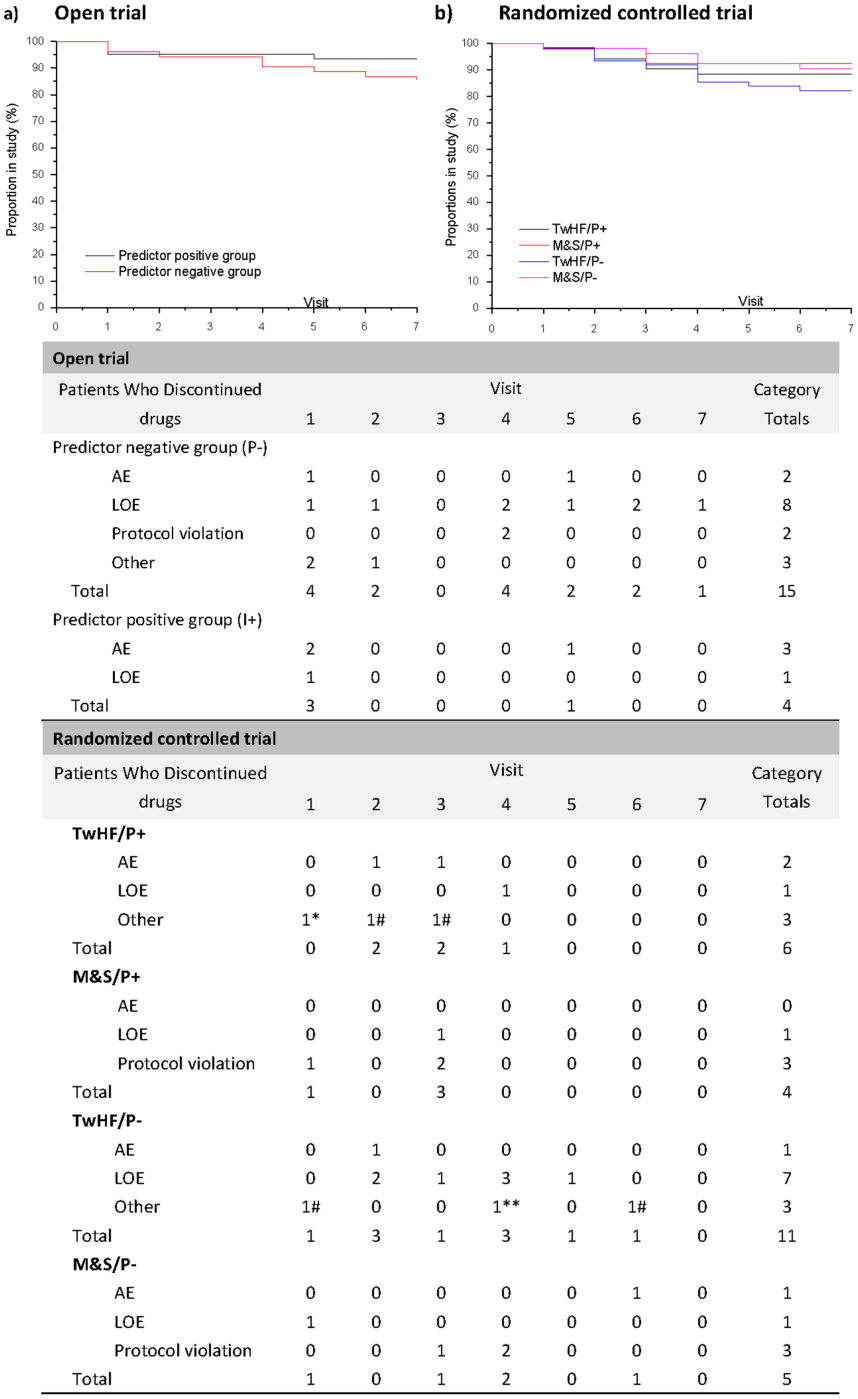
Time trajectory of withdrawals in two stages of the clinical trial. TwHF: TwHF based therapy group; M&S: MTX plus SSZ group; P+: Predictor positive group; P−: Predictor negative group. Values below the trajectory are the numbers of patients in the TwHF based therapy and MTX plus SSZ groups who discontinued treatment because of AEs, LOE, or other reasons. AE = adverse events; LOE = Lack of efficacy. *: leukopenia. **: complication. #: protocol violation.

A total of 148 patients completed the 24 week treatment in the open trial; 192 participants completed the study in the RCT. The study flow, the reasons for discontinuation, and the time trajectory of withdrawals of the study are shown in Figure 1 and 2.

### Baseline Characteristics of the Patients

Demographic characteristics at baseline are described in Table 1. There were no significant differences in baseline characteristics between TwHF/P+ vs M&S/P+, and TwHF/P− vs M&S/P− groups, except duration of disease and joint function classification between TwHF/P− and M&S/P− group in the RCT.

### Clinical Effectiveness

**Figure 3:**
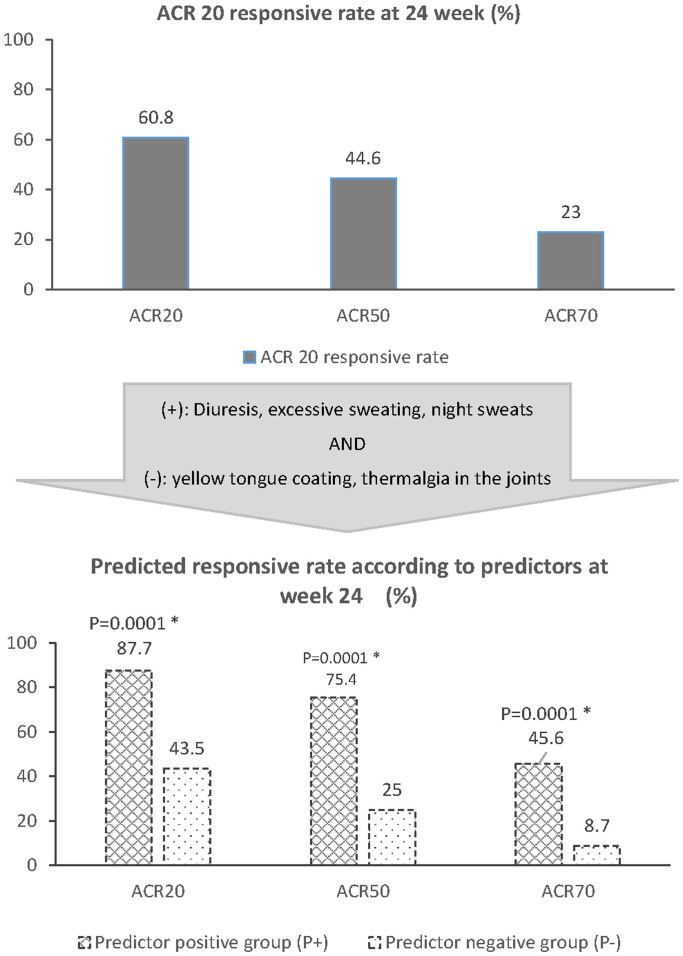
Effective rate according to the Criteria of the American College of Rheumatology (ACR) at 24 week. Upper part: ACR 20, 50, 70 responsive rates in the open trial. Middle part: The identified symptomatic predictors. A patient presented with any one of diuresis, excessive sweating, and night sweats, without any of yellow tongue coating and thermalgia in the joints would be classified into the predictor positive group (P+); otherwise, the patient would be classified into the predictor negative group (P−). Lower part: Predicted ACR 20, 50, 70 responsive rates in P+ and P− groups after the patients were classified based on the identified symptomatic predictors. There would be significant differences between P+ and P− groups in the ACR 20, 50, and 70 responsive rates (P < 0.005).

In the open trial, 60.8% of patients achieved ACR20 response after 24 week treatment (95% CI, 53.0% to 68.7%) among patients who completed the study. ACR50 and 70 responsive rates were 43.1% (59 cases) and 24.1% (33 cases), respectively (Fig. 3, upper part). The result was accessed as stable and credible comparing with the corresponding ACR20 responsive rate at 53.2% in TwHF based therapy in the previous randomized controlled study^24^,^35^,^40^.

Through univariate Chi Square analysis and multivariate analysis approaches PLS method (Supplementary Table 1 and 2), diuresis, excessive sweating, and night sweats were identified as the predictors which have positive correlation with response; yellow tongue coating, and thermalgia in the joints were as the predictors having inverse correlation with response. As described in Supplementary table 2, the diuresis means long voidings of clear urine; excessive sweating is described as excessive sweating during the daytime with no apparent cause such as physical exertion, hot weather, thick clothing or medication; night sweats means sweating during sleep that ceases on awakening; yellow tongue coating means there are a yellow colored coating in tongue; and thermalgia in the joints means local with burning sensation in joints.

In short, if a patient was presented with any one of diuresis, excessive sweating, and night sweats; AND without yellow tongue coating and thermalgia in the joints, then the patient was classified into the predictor positive group (P+); otherwise, the patient was classified into the predictor negative group (P−). Presumably, the ACR 20 responsive rate in the P+ group of patients in the open trial would be predicted to be 87.7%, and in the P− group would be at 43.5% (Fig. 3, lower part).

In the RCT, after 24 week treatment, ACR 20 responsive rates in TwHF/P+ group, M&S/P+ group, TwHF/P− group, M&S/P− group were 82.6% (38 from 46 patients), 64.6% (31 from 48), 52.9 (27 from 51), 85.1% (40 from 47), respectively. In P+ group of patients, TwHF based therapy showed significantly better effectiveness than M&S therapy (*P* < 0.05); reversely, in P− group, M&S indicated greater effectiveness than TwHF based therapy (*P* < 0.05). The RR value in P+ group based on ACR 20 response is 1.2791 (95% CI: 0.9982–1.639, P = 0.0492), which showed that, in the P+ group of patients, the ACR 20 responsive rate of TwHF based therapy was greater than M&S therapy; RRR value (RR in P+ group/RR in P− group) is 2.0563, and the 95% CI (1.4095–3) indicated the ACR 20 responsive rate of TwHF based therapy was better in P+ group than in P− (Table 2). Similar improvement in the ACR 50 response was observed, yet no significant difference was detected in ACR 70 response (Table 2).

The comparisons of the clinical responses in each item by ACR criteria and HAQ score at 24 week among groups are shown in Supplementary Figure 2.

### Use of Votalin

See Supplementary Table 3. There were no significant differences in Votalin usage between TwHF group and M&S group in both predictor positive and negative group (all P > 0.05).

### Safety

In both stages of the trial, the severity of most adverse events (AEs) was mild or moderate. There were no significant differences in most AEs between every two groups in the RCT. Incidence of AEs are presented in Table 3.

## Discussion

Our study identified the baseline symptomatic predictors of response to the TwHF based therapy in treating RA patients. The results indicates a huge potential of the TwFH based therapies in the value of treating the subpopulation of RA patients as an alternative therapy. Furthermore, the predicting ability of symptoms combination of response to therapy in RA was proved with RCT.

The first notable advantage of this study lies in the two-staged design, which, different from the retrospective analysis and single clinical trial, paying fully attention to the symptomatic predictors identification and verification in light of TCM experiences in two steps under a reasonable logical framework, which has been ignored in many clinical studies.

The process of comprehensive analysis of clinical information obtained by the TCM diagnostic procedures including observation, listening, questioning, and pulse analyses^16^. These baseline symptoms and signs are used as predictors of intervention selection, therapy outcomes and prognostic indicators in TCM for thousands of years, which are composed of multiple symptoms across the whole body, for example, the color of tongue coating, yet they are easy and intuitional to collect by inspection and inquiry, thus the use of them is convenient and affordable.

In the real practice of TCM, the pattern could be defined by the TCM doctors who make decision based on TCM pattern information; however the information might be slightly diversified among different TCM doctors^41^, and also it would be hard to be convincing for setting the information into the inclusion criteria in a RCT directly, which is recommended to establish the efficacy of TCM treatment^42^,^43^. In order to have convincing symptomatic predictor for a RCT based on TCM pattern classification, two-stage clinical trial is proposed^28^, and the convincing symptomatic predictors could be obtained in the first stage, open clinical trial.

Given there were as many as thirty symptom candidates, the determination of predictors was the crucial step in the whole study. The predictor analysis was divided to two steps. In the first step chi square test was used to screen the symptoms which was closely correlated with clinical response preliminarily as in many similar studies^22^,^23^. Then multivariate analysis procedure was utilized to narrow the number of predictors by obtaining a weight score for each parameter candidate as there are multiple parameters in analysis^19^,^20^. PLS method was employed as a variance based technique which is less sensitive to multicollinearity^44^,^45^. Most importantly, the final identified predictors were also approved by TCM clinicians based on TCM theory and clinical experiences, which ensured the fulfilling of the second stage trial.

Another strength of this study was the intervention selection for control group, the combination of MTX plus SSZ was selected. Previous studies tended to use DMARD monotherapy as the positive control drug, which can bring a higher possibility of positive outcomes, for the effectiveness of DMARDs monotherapy is usually lower than combination therapy^35^,^46^. Thus in the present trials, our results can ascertain the greater effectiveness of TwHF based therapy with higher power and practicality. In the TwHF based therapy, GTT, as one of the most frequently used TwHF products, has been extensively used in China for RA treatment. GTT have been shown to inhibit production of proinflammatory cytokines by monocytes and lymphocytes, as well as prostaglandin E2 production via the cyclooxygenase, COX-2, pathway, a potential mechanism of action in patients with RA^47^. In an expert consensus study, GTT was recommended by 85.7% of the clinical experts in China for the treatment of active RA^48^. YSJB pill is a potential anti-rheumatic agent targeting the inflammatory and immunomodulatory response of macrophages, it has been reported to be able to significantly decrease the production of peritoneal macrophages derived TNF-α, IL-1 and NO of serum, and decrease the TNF-alpha mRNA, IL-1beta mRNA, and caspase-3 expression in synoviocytes in Freund's complete induced adjuvant arthritis (AA) in rat model^49^,^50^. YSJB also has protective effect on the TCM kidney deficiency pattern induced by androgen deficiency in CIA rats^51^. Such characteristics of YSJB may be advantageous to the treatment of clinical RA with TCM deficiency pattern. In our trial, TwHF product is used with combination of YSJB, which is recommended as a therapy of RA in TCM clinical practice guideline for RA treatment. Pharmacologically, both GTT and YSJB have been shown to have anti-inflammatory and immune inhibitory effects for the treatment of RA animal models^47^,^50^,^51^, yet there was no study concentrate on the synergetic effect of YSJB with TwHF.

According to the TCM clinical practice, the patients with RA can be classified into two main patterns: the cold pattern and the hot pattern. The RA with cold pattern can be described as severe pain in a joint or muscle that limits the range of comfortable movement which does not move to other locations. The pain is relieved by applying warmth to the affected area, but increases with exposure to cold. Loose stools are characteristic as well as an absence of thirst and clear profuse urine. A thin white tongue coating is seen, combined with a wiry and tight pulse. In contrast, the RA with heat pattern is characterized by severe pain with hot, red, swollen and inflamed joints. Pain is generally relieved by applying cold to the joints. Other symptoms include fever, thirst, a flushed face, irritability, restlessness, constipation and deep-colored urine. The tongue may be red with a yellow coating and the pulse may be rapid^27^.

The identified symptomatic predictors for the TwHF based therapy included three symptoms with positive correlation (Diuresis, excessive sweating, night sweats) and two symptoms with negative correlation to effectiveness (yellow tongue coating, and thermalgia in the joints). The formers are frequently seen in the patients with TCM cold or deficiency pattern while both the latter symptoms belong to TCM hot pattern according to TCM pattern classification theory. Thus our results indicated the TwHF based therapy would have better effectiveness in patients with cold/deficiency pattern rather than patients with hot pattern, which was in accordance with traditional TCM cognition^31^.

Also interestingly, MTX plus SSZ therapy showed different effectiveness in different subgroups of patients defined with the predictors. This finding indicates a possible biological difference between the two subgroups of RA patients, although the underlying mechanism still remains unclear. However, the TwHF based therapy showed a higher effective rate in treating the RA patients who showed lower response to MTX plus SSZ combination therapy. This finding might be valuable and inspiring for identifying the specific responders for the therapy.

Actually, similar outcome (different effectiveness in different subgroups of MTX based therapy in RA patients based on Chinese symptoms) was achieved and published in our previous study^27^, the underlying mechanism was analyzed using bio-network based approaches^39^,^52^. To date, there has been several types of biomarkers being used in clinical setting, including proteins, specific variations in the DNA sequence, abnormal methylation patterns, aberrant transcripts, microRNAs, or other biological molecules, such as lipids and metabolic products, these biomarkers are used to assess the progress of some disease and treatment effect, or to estimate the risk of some disease such as cancer^53^. However, all these biomarkers are targeting to some specific disease, none of them can be used to elucidate the symptom or symptom combination. It has been demonstrated that in the context of TCM theory, network biology based approaches can prioritize disease-associated genes, predict the target profiles and pharmacological actions of herbal compounds, interpret the combinational rules and network regulation effects of herbal formulae^54^,^55^,^56^, thus the biomarkers for our symptomatic indicators might be a network based profile instead of a single molecule. The identification of such a network based profile will rely on a larger scale study, which is now developed underway as further study of this two-staged trial.

There are still some limitations in this study. Firstly, only 24 week therapy was conducted. Long-term data were not collected in the participants, and thus the differences among groups on healing of bone erosions and protective efficacy couldn't be observed. Secondly the symptomatic predictors based biomarkers are not yet identified because of its complexity. Finally the washout period of DMARDs was ever questioned as not long enough, yet after referring to some highly cited studies^36^,^57^, with a consideration for guarantee of the patient compliance, a longer washout period was denied for it might result in a higher difficulty in patient recruitment.

## Conclusions

The identified five symptomatic predictors enable the TwHF based therapy more efficiently in treating RA subpopulations. TwHF based therapy can effectively and safely treat the subgroup of active RA patient defined by the symptomatic predictors comparing with MTX plus SSZ combination therapy. This may assist to guide clinicians in making treatment decisions in clinical practice.

## Supplementary Material

Supplementary Information Supplementary Information

## Figures and Tables

**Table 1:** Patient Characteristics at Baseline in the two stages of the clinical trial

The fist-stage open trial *				The second-stage randomized controlled trial **			
Characteristics	Overall Population (N = 148)	Predictor positive population (n = 57)	Predictor negative population (n = 91)	TwHF/P+ group (n = 46)	M&S/P+ group (n = 48)	TwHF/P− group (n = 51)	M&S/P− group (n = 47)
Age (yr)							
Mean (SD)	48.23 (10.81)	49.91 (8.86)	47.18 (11.79)	46.72 (10.71)	47.47 (9.94)	45.48 (11.74)	48.06 ± 10.88
Range	19.00–65.00	24.00–65.00	19.00–65.00	22.62–69.97	29.60–68.00	21.65–68.00	22.97–70.74
Gender (F/M)	121/27	49/8	72/19	39/7	37/11	40/11	39/8
Source (Outpatients/Inpatients)	143/5	55/2	88/3	44/2	46/2	47/4	43/4
Duration of disease (month)∥	49.78 (62.14)	35.09 (55.23)	58.99 (64.70)	89.22 (87.49)	81.71 (81.74)	56.24 (42.97)	85.17 (79.79) *
Comorbidities (Without/With)	121/27	50/7	71/20	43/3	39/9	49/2	43/4
Positive serum test for rheumatoid factor n (%)	112 (78.9%)	43 (76.8)	69 (80.2)	89.1	93.6	85.4	91.5
Joint function classification n (%)∥							
I	6 (4.1)	1 (1.8)	5 (5.5)	1 (2.2)	1 (2.1)	8 (15.7)	2 (4.3)
II	86 (58.1)	19 (33.3)	67 (73.6)	28 (60.9)	31 (64.6)	32 (62.8)	25 (53.2)
III	56 (37.8)	37 (64.9)	19 (20.9)	17 (37.0)	16 (33.3)	11 (21.6)	20 (42.6)
No. of tender joints †	15.61 (8.4)	21.40 (7.9)	11.99 (6.6)	22.07 (10.19)	17.94 (9.93)	14.24 (7.99)	16.49 (8.71)
No. of swollen joints†	11.06 (6.5)	14.14 (6.4)	9.13 (5.8)	14.09 (8.57)	11.33 (7.09)	9.76 (6.30)	10.28 (6.33)
Rest pain	58.82 (22.33)	70.00 (19.59)	51.81 (21.13)	58.48 (22.99)	56.35 (21.33)	48.00 (25.88)	49.36 (26.24)
Early morning stiffness	96.82 (78.92)	113.5 (80.27)	86.37 (76.66)	85.26 (61.01)	95.77 (69.35)	82.45 (58.32)	87.70 (100.31)
Patients overall assessment of disease activity—VAS (mm) ‡	65.70 (18.98)	73.42 (18.06)	60.86 (18.01)	65.22 (16.02)	60.56 (16.08)	57.84 (19.32)	63.30 (±20.12)
Doctors overall assessment of disease activity—VAS (mm) ‡	62.64 (18.68)	69.32 (15.95)	58.45 (19.12)	62.83 (15.30)	58.33 (15.34)	54.51 (18.47)	59.79 (20.05)
Health assessment questionnaire score (HAQ) §	7.38 (5.83)	11.11 (6.26)	5.04 (4.12)	7.74 (5.48)	6.54 (4.73)	5.65 (4.56)	6.79 (4.80)
ESR (mm/h)	44.50 (29.65)	45.20 (33.45)	44.07 (27.21)	50.48 (27.17)	43.63 (22.06)	45.16 (23.12)	48.89 (29.26)
CRP (ng/L)	12.46 (18.22)	5.64 (9.40)	16.71 (20.95)	18.56 (28.25)	20.09 (31.47)	22.19 (26.46)	20.71 (24.05)

CRP = C-reactive protein; ESR = erythrocyte sedimentation rate.

*Numeral values followed by sign of aggregation are means (SDs). There were no significant difference in any of the characteristics between the predictor positive population and the predictor negative population.

**Numeral values followed by sign of aggregation are means (SDs). There were no significant difference in any of the characteristics between the TwHF based therapy and MTX plus SSZ group except the duration of disease between TwHF/P− and M&S/P− group (*P* = 0.0455).

†Forty joints were assessed for swelling and tenderness.

‡Patients and Doctors overall assessment of disease activity were assessed with the use of visual-analogue scales (VAS), scores can range from 0 to 100 mm, with higher scores indicating poorer status or more severe disease activity.

§HAQ contains 8 items and the score of each item can range from 0 (no difficulty) to 3 (unable to perform the activity). Lower scores indicate a better quality of life.

∥There was significant difference between TwHF/P− and M&S/P− group, for duration of disease, the F value = 4.12, P = 0.045; for joint function classification, Z Wilcoxon = 2.8, P = 0.0098.

**Table 2:** ACR responsive rate at 24 week and the comparisons between the predictor positive and predictor negative group

Group		ACR 20				ACR 50				ACR 70			
		P+ group		P− group		P+ group		P− group		P+ group		P− group	
		n	Responsive rate n (%)	n	Responsive rate n (%)	n	Responsive rate n (%)	n	Responsive rate n (%)	n	Responsive rate n (%)	n	Responsive rate n (%)
**TwHF based therapy group**		46	38 (82.6)	51	27 (52.9)	46	26 (56.5)	51	11 (21.6)	46	6 (13.0)	51	6 (11.8)
**MTX plus SSZ group**		48	31 (64.6)	47	40 (85.1)	48	22 (45.8)	47	28 (59.6)	48	12 (25.0)	47	13 (27.7)
Chi Square		3.91		11.7		1.07		14.75		2.17		3.95	
*P* value		0.048 *		<0.001 *		0.300		<0.001 *		0.141		0.047 *	
**RR** †	RR (TwHF/M&S)	1.2791		0.6221		1.2332		0.362		0.5217		0.4253	
	95%CI for RR	0.9982–1.639		0.4678–0.8272		0.8279–1.837		0.2039–0.6427		0.2137–1.2739		0.176–1.0279	
	MH Χ^2^	3.8678		11.581		1.0625		14.5957		2.1459		3.9136	
	*P* value	0.049		<0.001 *		0.303		<0.001 *		0.143		0.048 *	
**Test of interaction**	Z value	3.741				3.4381				0.319			
	*P* value	<0.001 *				<0.001 *				0.375			
**RRR** ‡	RRR	2.0563				3.4065				1.2267			
	95%CI for RRR	1.4095–3				1.6938–6.851				0.3496–4.3038			

*P<0.05.

†RR: Relative risks = Responsive rate in TwHF based group/Responsive rate in MTX plus SSZ group.

‡RRR: Ratio of relative risks = RR in predictor positive group/RR in predictor negative group.

The RR value in predictor positive group based on ACR 20 response = 1.2791 (95% CI: 0.9982–1.639, P = 0.0492), which indicates that in the predictor positive group of patients, the ACR 20 responsive rate of CHM is greater than MTX plus SSZ; the RRR value = 2.0563 > 1, and the 95% CI (1.4095–3) doesn't include 1, which indicate that the ACR 20 responsive rate of TwHF is better in predictor positive group than in predictor negative group.

**Table 3:** Summary of adverse events in the two stages of the clinical trial

Summary of adverse events in the open trial		
Variable	N = 167 n (%)	AEs leading to discontinuation of drug
Any adverse events	53 (31.7)	5
Hepatic dysfunction		
Alanine aminotransferase elevation	21 (12.6)	0
Aspartate aminotransferase elevation	21 (12.6)	0
Erythra, Pruritus	3 (1.8)	2
Drug allergy	2 (1.2)	0
Leukopenia	1 (0.6)	1
Gastrointestinal reaction	1 (0.6)	1
Anorexia	1 (0.6)	0
Amenorrhoea	1 (0.6)	0
Renal function abnormal	1 (0.6)	0
Fractures	1 (0.6)	1∥
**Serious adverse events, possibly related to study drug** ^$^		
Acute hepatic dysfunction	1 (0.6)	1
Anorexia	1 (0.6)	0
Renal function abnormal	1 (0.6)	0

Summary of adverse events in the randomized controlled trial n (%)*				
Variable	TwHF/P+ (N = 52)	M&S/P+ (N = 53)	TwHF/P− (N = 62)	M&S/P− (N = 51)
Cases with AEs n (%)	14 (26.9)	19 (35.9)	16 (25.2)	13 (25.5)
Any adverse event				
Alanine aminotransferase elevation	5 (9.6)	10 (18.9)	9 (14.5)	6 (11.8)
Aspartate aminotransferase elevation	6 (11.5)	6 (11.3)	4 (6.5)	5 (9.8)
Hepatic dysfunction	3 (5.8)	4 (7.6)	5 (8.1)	1 (2.0)
Gastrointestinal reaction	1 (1.9)	6 (11.3)	2 (3.2)	4 (7.84)
Cough	3 (5.8)	1 (1.9)	0	0
Leukopenia	1 (1.9)	0	0	2 (3.92)
Positive urinary occult blood	0	1 (1.9)	2 (3.2)	1 (2.0)
Dizziness	0	1 (1.9)	0	1 (2.0)
Amenorrhoea	0	1 (1.9)	0	0
Cholelithiasis	0	1 (1.9)	0	0
Multiple organ failure (MOF)	0	0 (0.0)	1 (1.6)	0
Common cold	0	1 (1.9)	0	0
Thyromegaly	0	1 (1.9)	0	0
Pruritus	1 (1.9)	0	0	0
Upper respiratory tract infection	1 (1.9)	0	0	0
Alimentary tract hemorrhage	1 (1.9)	0	0	0
Eyes blurred, muscae volitantes	0	1 (1.9)	0	0
Cerebrovascular accident	0	0	1 (1.6)	0
Uric acid elevation	0	0	1 (1.6)	0
Positive urinary ketone	0	0	0	1 (2.0)
Abnormal blood routine examination	0	0	1 (1.6)	0
Blood platelets elevation	0	0	1 (1.6)	0
Numbness in the left hand	0	0	0	1 (2.0)
Surgery for ganglion of the left wrist	0	0	0	1 (2.0)
**Serious adverse events, possibly related to study drug** ^$^	2 (3.84)	2 (3.78)	4 (6.45)	1 (1.96)
Acute hepatic function damage	1 (1.9)	0	0	0
Amenorrhoea	0	1 (1.9)	0	0
Leukopenia	1 (1.9)	0	0	0
Gastrointestinal reaction	0	0	0	0
Hepatic dysfunction	0	1 (1.9)	1 (1.6)	1 (2.0)
Multiple organ failure (MOF)	0	0	1 (1.6)	0
Uric acid elevation	0	0	1 (1.6)	0
Abnormal blood routine examination	0	0	1 (1.6)	0
**Adverse events leading to discontinuation of drug**	4 (7.7)	2 (3.7)	3 (4.8)	1 (2.0)
Acute hepatic function damage	1 (1.9) ** ‡‡	0	1 (1.6) ¶ ††	1 (2.0) ¶ ‡‡
Alimentary tract hemorrhage	1 (1.9) † ††	0	1 (1.6) † ‡‡	0
Multiple organ failure (MOF)	0	0	1 (1.6) ‡ ‡‡	0
Gastrointestinal reaction	0	1 (1.9) || ††	0	0
Leukopenia	1 (1.9) ¶ ††	0	0	0
Pruritus	1 (1.9) || ††	0	0	0
Cholelithiasis	0	1 (1.9) † ‡‡	0	0

*n was the number of recorded AEs. There were no significant differences in all AEs between TwHF/P+ and M&S/P+ group and between TwHF/P− and M&S/P− group. The n in Cases with AEs item represents the cases of patients who suffered at least one AE, one patient could suffer more than one AE.

$Serious adverse events were death or any event that was life-threatening; required hospitalization or prolongation of existing hospitalization; resulted in persistent or significant disability, congenital anomaly, or spontaneous or elective abortion; or required medical or surgical intervention to prevent another serious outcome.

In the open trial, in addition to the serious adverse events listed, fractures in 1 patient also occurred but were not considered to be possibly related to the study drug.

In the randomized controlled trila, in addition to the serious adverse events listed, the following serious adverse events also occurred (in 1 patient each) but were not considered to be possibly related to the study drug: cerebrovascular accident, Thyromegaly, cholelithiasis, Alimentary tract hemorrhage.

†This AE was considered to be unlikely related to the study drug.

‡This AE was considered to be suspiciously related to the study drug and the symptom released after discontinuation of study drug.

∥This AE was considered to be possibly related to the study drug and the symptom released soon after discontinuation of the drugs.

¶This AE was considered to be probably related to the study drug and the symptom released soon after discontinuation of the drugs.

**This AE was considered to be positively related to the study drug and the symptom released soon after discontinuation of the drugs.

††The symptom continued after discontinuation of study drug without life-threatening events.

‡‡The symptom released after discontinuation of study drug.
