# Supplementary material for: Predicting and verifying outcome of Tripterygium wilfordii Hook F. based therapy in rheumatoid arthritis: from open to double-blinded randomized trial
Source: Sci Rep. 2015 Apr 15;5:9700. doi: 10.1038/srep09700 (PMC5155630; doi:10.1038/srep09700)

**Predicting and verifying outcome of *Tripterygium wilfordii Hook F.* based therapy in rheumatoid arthritis: from open to double-blinded randomized trial**

*Corresponding Author

Aiping Lu, Dongzhimennei Nanxiaojie No.16, Phone +861064067611, Fax + 861084032881; E-mail [aipinglu@hkbu.edu.hk](mailto:aipinglu@hkbu.edu.hk).

Miao Jiang ^1,2#^, Qinglin Zha ^3#^, Chi Zhang ^1^, Cheng Lu ^1^, Xiaoping Yan ^4^, Wanhua Zhu ^5^ , Wei Liu ^6^, Shenghao Tu ^7^, Liping Hou ^8^, Chengwu Wang ^9^, Wandong Zhang ^10^, Qinghua Liang ^11^, Bing Fan ^12^, Jiangping Yu ^13^, Weidong Zhang ^14^, Xinru Liu ^14^, Jing Yang ^1^, Xiaojuan He ^1^, Li Li ^1^, Xuyan Niu ^1^, Yan Liu ^1^, Hongtao Guo ^15^, Bing He ^2^, Ge Zhang ^2^, Zhaoxiang Bian ^2^, Aiping Lu ^1,2 *^

1. Institute of Basic Research in Clinical Medicine, China Academy of Chinese Medical Sciences, Beijing 100700, P.R. China.
2. School of Chinese Medicine, Hong Kong Baptist University, Kowloon Tong, Kowloon 999077, Hong Kong
3. School of Computer, Jiangxi University of Traditional Chinese Medicine, Nanchang 330004, P.R. China
4. Department of Rheumatism, Beijing China-Japan Hospital, Beijing 100621, P.R. China.
5. Nantong Liangchun Rheumatology Hospital, Nantong, Jiangsu 226009, P.R. China.
6. Department of Rheumatism, The first affiliated hospital of Tianjin University of TCM, Tianjin 300193, P.R. China.
7. Department of Rheumatism, Hubei Tongji Hospital, Wuhan, Hubei 430030, P.R. China.
8. Shanxi Taiyuan Rheumatology Hospital, Taiyuan 030006, Shanxi, P.R. China.
9. Department of Rheumatism, The first hospital affiliated to Jilin Changchun University of TCM, Changchun, Jilin 130021, P.R. China.
10. Department of Rheumatism, The first hospital affiliated to Anhui University of TCM, Hefei, Anhui 230031, P.R. China.
11. Department of Rheumatism, Hunan Xiangya Hopital, Changsha, Hunan 410008, P.R. China.
12. Department of Rheumatism, Affiliated Hospital to Shandong University of TCM, Jinan, Shandong 250011, P.R. China.
13. Department of Rheumatism, The first hospital affiliated to Jiangxi University of TCM, Nanchang 330020, Jiangxi, P.R. China.
14. College of Pharmacy, The Second Military Medical University, Shanghai 200433, P.R. China.
15. Department of Rheumatism, The first hospital affiliated to Henan College of Chinese medicine, Zhengzhou, Henan, 450000, P.R. China.

Author Contributions

# These authors contributed equally to this work.

Notes

The authors declare no competing financial interest.

**Supplementary materials**

| **Supplementary Table 1 - Symptomatic predictors according to ACR 20 response** | | | | | | | |
| --- | --- | --- | --- | --- | --- | --- | --- |
| **No.** | **SYMPTOM (Lable)** | **Category** | **Effective case no (%)** | **Non-effective case no (%)** | **Univariate Chi Square** | **P value** | **PLS Weight** |
|  | Diuresis * | Without | 60 (51.7) | 56 (48.3) | 18.59 | <0.001 | 0.34 |
|  |  | With | 30 (93.8) | 2 (6.3) |  |  |  |
|  | Chilly | Without | 22 (59.5) | 15 (40.5) | 0.04 | 0.846 | - |
|  |  | With | 68 (61.3) | 43 (38.7) |  |  |  |
|  | Cold limbs | Without | 41 (52.6) | 37 (47.4) | 4.71 | 0.030 | 0.11 |
|  |  | With | 49 (70.0) | 21 (30.0) |  |  |  |
|  | Pale tongue | Without | 53 (63.1) | 31 (36.9) | 0.43 | 0.514 | - |
|  |  | With | 37 (57.8) | 27 (42.2) |  |  |  |
|  | White tongue-coating | Without | 31 (59.6) | 21 (40.4) | 0.05 | 0.827 | - |
|  |  | With | 59 (61.5) | 37 (38.5) |  |  |  |
|  | Pale white complexion | Without | 65 (58.0) | 47 (42.0) | 1.49 | 0.223 | - |
|  |  | With | 25 (69.4) | 11 (30.6) |  |  |  |
|  | Fear of cold in joints | Without | 20 (52.6) | 18 (47.4) | 1.44 | 0.231 | - |
|  |  | With | 70 (63.6) | 40 (36.4) |  |  |  |
|  | Fever | Without | 90 (62.5) | 54 (37.5) | 4.03 (Adj. ChiSQ.) | 0.0458 | -0.12 |
|  |  | With | 0 (0.0) | 4 (100.0) |  |  |  |
|  | Thermalgia in the joints * | Without | 51 (66.2) | 26 (33.8) | 1.98 | 0.159 | -0.33 |
|  |  | With | 39 (54.9) | 32 (45.1) |  |  |  |
|  | Yellow tongue-coating * | Without | 70 (64.2) | 39 (35.8) | 2.02 | 0.156 | -0.22 |
|  |  | With | 20 (51.3) | 19 (48.7) |  |  |  |
|  | Red tongue | Without | 55 (57.9) | 40 (42.1) | 0.95 | 0.331 | - |
|  |  | With | 35 (66.0) | 18 (34.0) |  |  |  |
|  | Yellow and turbid urine | Without | 74 (61.2) | 47 (38.8) | 0.03 | 0.855 | - |
|  |  | With | 16 (59.3) | 11 (40.7) |  |  |  |
|  | Obstipation | Without | 79 (60.3) | 52 (39.7) | 0.12 | 0.727 | - |
|  |  | With | 11 (64.7) | 6 (35.3) |  |  |  |
|  | Night sweats * | Without | 51 (51.0) | 49 (49.0) | 11.93 | <0.001 | 0.12 |
|  |  | With | 38 (80.9) | 9 (19.2) |  |  |  |
|  | Excessive sweating * | Without | 46 (49.5) | 47 (50.5) | 13.52 | <0.001 | 0.35 |
|  |  | With | 44 (80.0) | 11 (20.0) |  |  |  |
|  | Soreness and weakness in the knees and lower back | Without | 18 (46.2) | 21 (53.9) | 4.77 | 0.029 | 0.11 |
|  |  | With | 72 (66.1) | 37 (33.9) |  |  |  |
|  | Bloated epigastrium and abdomen | Without | 75 (57.7 | 55 (42.3) | 4.36 | 0.037 | 0.22 |
|  |  | With | 15 (83.3) | 3 (16.7) |  |  |  |
|  | Enlarged tongue | Without | 77 (63.6) | 44 (36.4) | 2.22 | 0.136 | -0.14 |
|  |  | With | 13 (48.2) | 14 (51.9) |  |  |  |
|  | Lassitude | Without | 34 (58.6) | 24 (41.4) | 0.19 | 0.661 | - |
|  |  | With | 56 (62.2) | 34 (37.8) |  |  |  |
|  | Torpid intake | Without | 71 (60.7) | 46 (39.3) | 0 | 0.951 | - |
|  |  | With | 19 (61.3) | 12 (38.7) |  |  |  |
|  | Absence of sweating | Without | 89 (63.1) | 52 (36.9) | 4.78 (Adj. ChiSQ.) | 0.029 | -0.24 |
|  |  | With | 1 (14.3) | 6 (85.7) |  |  |  |
|  | Numbness in the extremities | Without | 47 (58.0) | 34 (42.0) | 0.58 | 0.445 | - |
|  |  | With | 43 (64.2) | 24 (35.8) |  |  |  |
|  | Nocturia | Without | 47 (66.2) | 24 (33.8) | 1.66 | 0.197 | -0.14 |
|  |  | With | 43 (55.8) | 34 (44.2) |  |  |  |
|  | Dizziness | Without | 33 (34.4) | 33 (34.4) | 2.66 | 0.103 | -0.13 |
|  |  | With | 27 (51.9) | 25 (48.1) |  |  |  |
|  | Chest distress | Without | 69 (57.5) | 51 (42.5) | 2.92 | 0.088 | 0.10 |
|  |  | With | 21 (75.0) | 7 (25.0) |  |  |  |
|  | Insomnia | Without | 51 (59.3) | 35 (40.7) | 0.2 | 0.658 | - |
|  |  | With | 39 (62.9) | 23 (37.1) |  |  |  |
|  | Inhibited bending and stretching in joints | Without | 3 (75.0) | 1 (25.0) | 0 (Adj. ChiSQ.) | 0.944 | - |
|  |  | With | 87 (60.4) | 57 (39.6) |  |  |  |
|  | Arthralgia | Without | 0 (0.0) | 0 (0.0) | 0.35 | 0.556 | - |
|  |  | With | 90 (60.8) | 58 (39.2) |  |  |  |
|  | Swelling joint | Without | 0 (0.0) | 1 (100.0) | Exact | 0.392 | - |
|  |  | With | 90 (61.2) | 57 (38.8) |  |  |  |
|  | Tender joint | Without | 0 (0.0) | 0 (0.0) | 0.43 | 0.514 | - |
|  |  | With | 90 (60.8) | 58 (39.2) |  |  |  |

*** Symptoms selected as predictors.**

**Supplementary Table 2 – Interpretation and definition of the Baseline Symptoms**

| **No.** | **Baseline symptoms** | **Definition/Description ^#^** | **In Chinese** |
| --- | --- | --- | --- |
| 1 | Diuresis * | Long voidings of clear urine | XIAO BIAN QING CHANG |
| 2 | Chilly | Sensation of cold which can be relieved by warmth, the same as intolerance of cold | WEI HAN |
| 3 | Cold limbs | Lack of warmth in the extremities | SHOU ZU BU WEN |
| 4 | Pale tongue | A tongue less red than normal, indicating qi and blood deficiency or presence of deficiency-cold | SHE DAN |
| 5 | White tongue-coating | Tongue coating white in color | TAI BAI |
| 6 | Pale white complexion | A colorless complexion, often indicating blood deficiency or profuse loss of blood | MIAN SE DAN BAI |
| 7 | Fear of cold in joints | Pain in joints of limbs which is aggravated by cold | GUAN JIE PA LENG |
| 8 | Fever | Elevation of the body temperature above the normal or subjective feeling of feverishness | FA RE |
| 9 | Thermalgia in the joints * | Local with burning sensation in joints | GUAN JIE FA RE |
| 10 | Yellow tongue-coating * | Tongue coating yellow in color | TAI HUANG |
| 11 | Red tongue | A tongue redder than normal, indicating presence of heat | SHE HONG |
| 12 | Yellow and turbid urine | A urine discoloration described as yellow and cloudy. | XIAO BIAN HUANG ZHUO |
| 13 | Obstipation | Hardened feces difficult to evacuate | BIAN JIE |
| 14 | Night sweats * | Sweating during sleep that ceases on awakening | DAO HAN |
| 15 | Excessive sweating * | Excessive sweating during the daytime with no apparent cause such as physical exertion, hot weather, thick clothing or medication | ZI HAN |
| 16 | Soreness and weakness in the knees and lower back | Limp aching lumbus and knees | YAO XI SUAN RUAN |
| 17 | Bloated epigastrium and abdomen | Bloating of the stomach duct and abdomen | WAN FU ZHANG MAN |
| 18 | Enlarged tongue | Enlarged tongue | SHE ZHI PANG |
| 19 | Lassitude | Lack of mental vigor | JING SHEN PI FA |
| 20 | Torpid intake | Loss of appetite with no desire for food with decreased intake, the same as poor appetite | NA DAI |
| 21 | Absence of sweating | Abnormal deficiency or absence of sweating, the same as anhidrosis | WU HAN |
| 22 | Numbness in the extremities | Reduced sensitivity to touch | ZHI TI MA MU |
| 23 | Nocturia | Profuse urination at night | YE NIAO DUO |
| 24 | Dizziness | A diseased state characterized by a whirling sensation in the head with visual distortion | XUAN YUN |
| 25 | Chest distress | Feeling of oppression in the chest | XIONG MEN |
| 26 | Insomnia | Prolonged inability to obtain normal sleep | SHI MIAN |
| 27 | Inhibited bending and stretching in joints | Inhibited bending and stretching in joints | GUAN JIE QU SHEN BU LI |
| 28 | Arthralgia | Joint pain | GUAN JIE TENG TONG |
| 29 | Swelling joint | The buildup of fluid in the soft tissue surrounding the joint | GUAN JIE ZHONG ZHANG |
| 30 | Tender joint | Joint tenderness when you press around the joint | GUAN JIE YA TONG |

**^#^** The Definition/Description of the symptoms and signs were referring to *Traditional Chinese Medicine Dictionary (In Chinese, People’s Health Press, 2005), the English-Chinese Chinese-English Dictionary of Chinese Medicine (Hunan Science & Technology Press, 1995)* and *WHO international standard terminologies on traditional medicine in the western pacific region (World Health Organization, 2007).*

**Supplementary Table 3 – Votalin** medication and comparisons between groups: n (%)

| **Group** | **Use of Votalin** | **Predictor positive group** | **Predictor negative group** | **Chi Square** | ***P* value** |
| --- | --- | --- | --- | --- | --- |
| Open trial | No use | 54 (94.7) | 75 (82.4) | 4.75 | 0.029 |
|  | Use | 3 (5.3) | 16 (17.6) |  |  |
| RCT | **Use of Votalin** | **TwHF group** | **M&S group** | **Chi Square** | ***P* value** |
| Predictor positive group | No use | 32 (69.6) | 35 (72.9) | 0.13 | 0.720 |
|  | Use | 14 (30.4) | 13 (27.1) |  |  |
| Predictor negative group | No use | 26 (51.0) | 30 (63.8) | 1.65 | 0.199 |
|  | Use | 25 (49.0) | 17 (36.2) |  |  |

**Supplementary Figure Legends**

**Supplementary Figure 1. Finger printings of different batches of YSJB**

**Upper part: standard fingerprint of YSJB from pharmacopoeias; medial part: finger prints of different 12 batches of marketed YSJB; lower table: semblance analysis between different batches of YSJB products The correlation coefficient of similarity of chromatographic fingerprints from each of the 12 batches of YSJB pills comparing with standard fingerprint was greater than 0.95, which indicated a high similarity among the 12 batches of YSJB and a desirable drug quality.**

**Supplementary Figure 2. Comparisons of clinical responeses by American Colledge of Rheumatology criteria and HAQ score at 24 week**

**TwHF: TwHF based therapy group; M&S: MTX plus SSZ group; P+: Predictor positive group; P-: Predictor negative group. ESR: erythrocyte sedimentation rate; CRP: C-reactive protein; HAQ: health assessment questionnaire; _20: 20% improvement of the item; _50: 50% improvement of the item; _70: 70% improvement of the item.**

**The figures in columns showed the numbers (in percentage form) of patients who achieved 20% (or 50%, 70%) improvement of the item.**

**#, ∆, € , ₫: Significant difference was detected in the 20% improvement of the item between correlated two groups; double symbols: significant difference in the 50% improvement of the item between correlated two groups; three symbols: significant difference in the 70% improvement of the item between correlated two groups (P<0.05).**

**#: TwHF/P+ group vs TwHF/P- group; ∆: M&S/P+ group vs M&S/P- group; €: TwHF/P+ group vs M&S/P+ group; ₫: TwHF/P- group vs M&S/P- group.**

**In the comparison between TwHF/P+ vs TwHF/P- group, there were significant differences (P<0.05) in swollen joints 20 (20% improvement, similarly hereinafter), swollen joints 50, tender joints 20 and 50, patient global score 20 and 50 and 70, HAQ score 20, and CRP 70. Comparing M&S/P+ group with M&S/P- group, significant difference (P<0.05) was detected only in tender joints 20. Comparing TwHF/P+ group with M&S/P+ group, significant differences (P<0.05) were detected in tender joints 20 and HAQ score 50. When comparing TwHF/P- group with M&S/P- group, significant differences (P<0.05) were identified in swollen joints 20 and 50, tender joints 20 and 50, patient global score 20 and 50, physician global score 20 and 50 and 70, ESR 20, and CRP 70.**

**Supplementary Figure 1. Finger printings of different batches of YSJB**

**
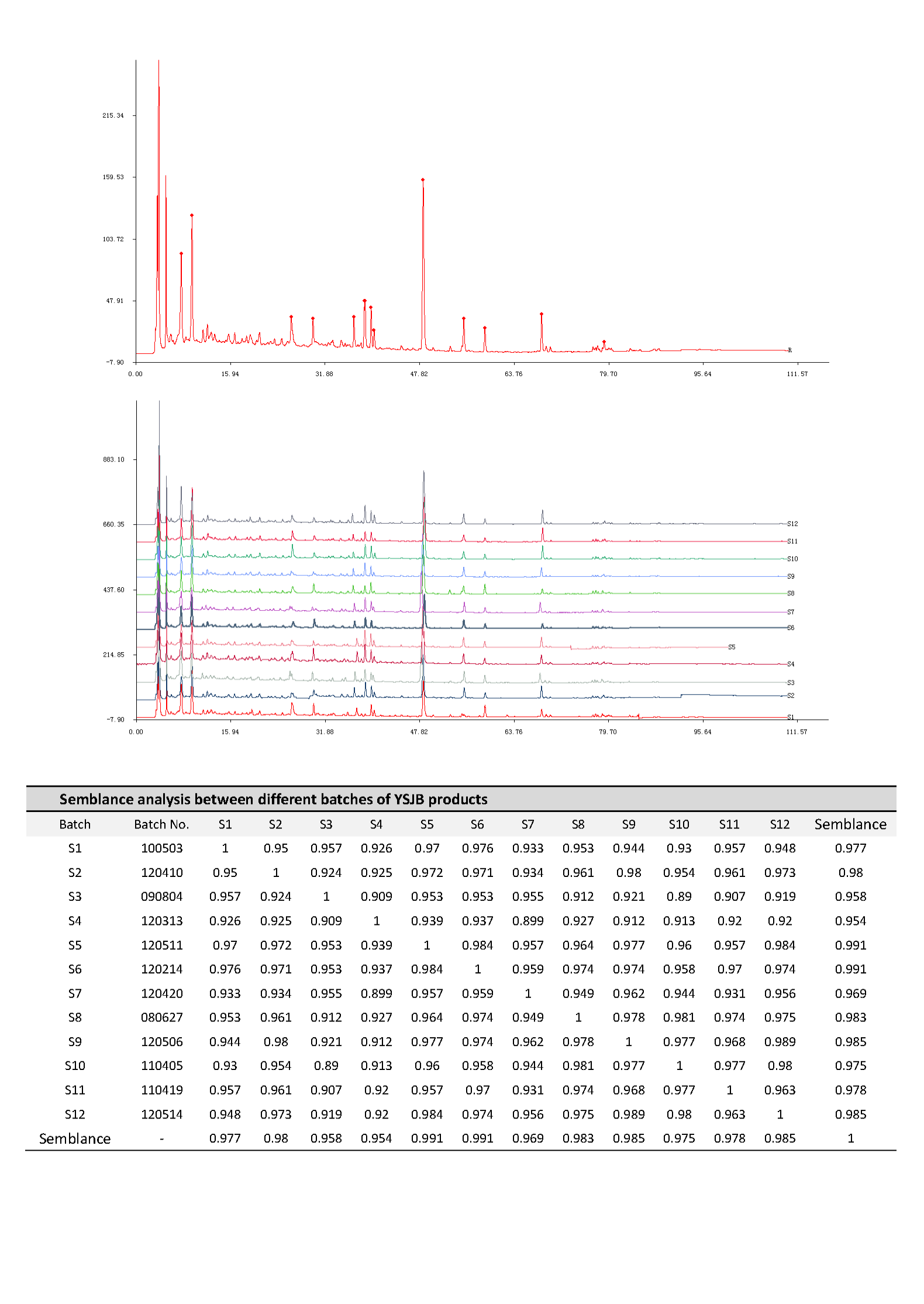
**

**Supplementary Figure 2. Comparisons of clinical responeses by American Colledge of Rheumatology criteria and HAQ score at 24 week**


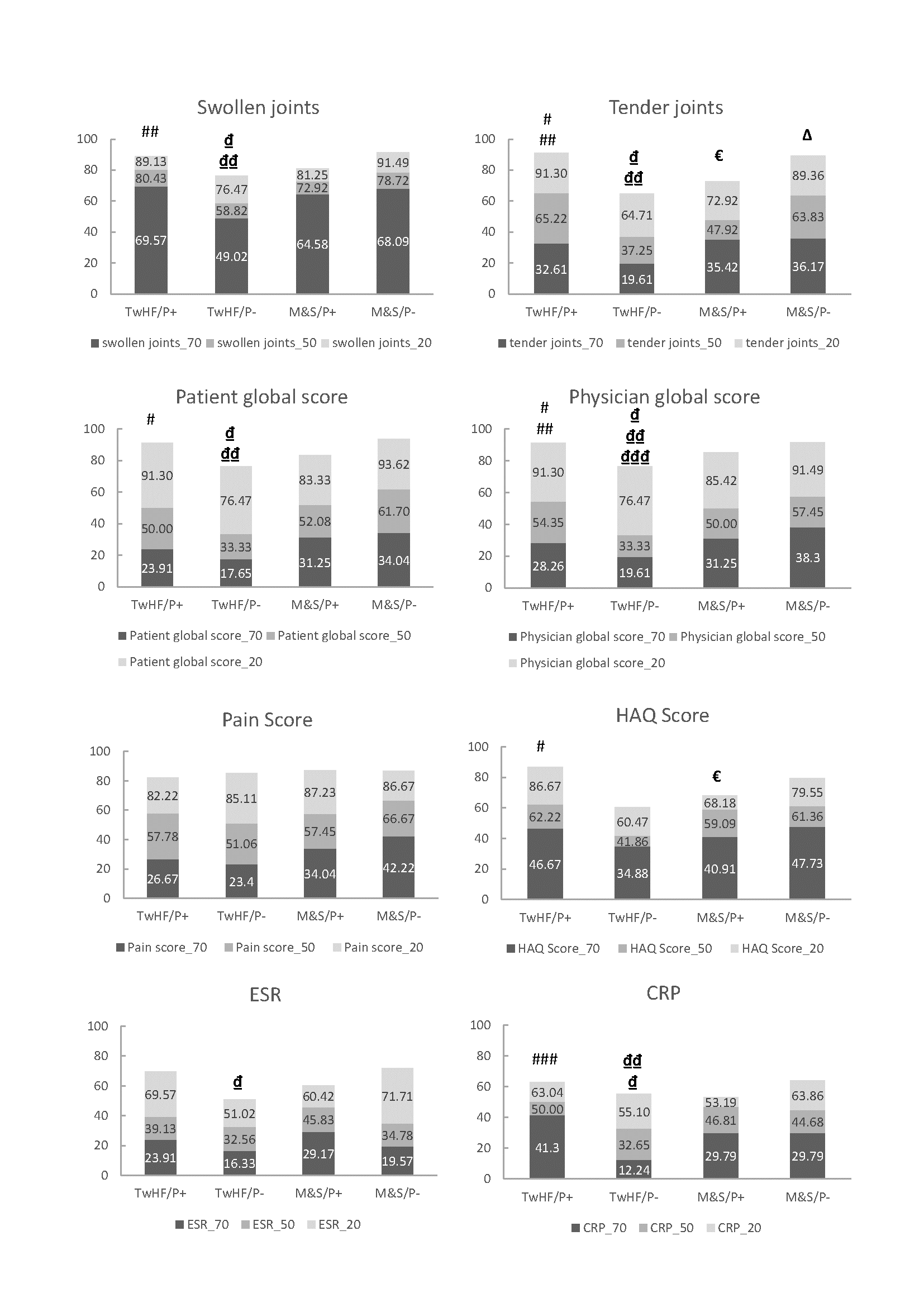

Supplement: Supplementary Information [file srep09700-s1.doc]
